# Asthma-COPD overlap is not a homogeneous disorder: further supporting data

**DOI:** 10.1186/s12931-017-0667-x

**Published:** 2017-11-02

**Authors:** Luis Pérez-de-Llano, Borja G. Cosio, on behalf of the CHACOS study group, Amanda Iglesias, Natividad de las Cuevas, Juan Jose Soler-Cataluña, Jose Luis Izquierdo, Jose Luis López-Campos, Carmen Calero, Vicente Plaza, Marc Miravitlles, Alfons Torrego, Eva Martínez Moragón, Joan B. Soriano, Antolin Lopez Viña, Irina Bobolea

**Affiliations:** 1 Department of Respiratory Medicine, Hospital Lucus Augusti, Lugo, Spain; 2 Department of Respiratory Medicine, Hospital Universitario Son Espases-IdISBa, Palma de Mallorca, Spain; 3 CIBER de Enfermedades Respiratorias (CIBERES), Instituto de Salud Carlos III, Madrid, Spain; 4 Pneumology Service, Hospital Universitario Lucus Augusti, Calle Dr Ulises Romero n° 1. 27003, Lugo, Spain

**Keywords:** Eosinophils, Periostin, COPD, Asthma, Asthma-COPD overlap, ACO

## Abstract

Asthma-COPD ovelap (ACO) is an umbrella term that encompasses patients with COPD and eosinophilic inflammation (e-COPD) and smoking asthmatics with non-fully reversible airflow obstruction (SA). We compared the clinical characteristics and the inflammatory profile of e-COPD and SA. Patients classified as e-COPD were older and more often male and showed significantly impaired pulmonary function (likely explained by a heavier smoking habit). On the contrary, SA had more atopic features, more reversibility of airflow obstruction and higher IgE levels. The concentrations of IL-5, IL-13, IL-8, IL-6, TNF-α, IL17 in serum were similar between the 2 groups. However, Th2-related biomarkers (periostin, FeNO and blood eosinophils) shower higher median values in e-COPD patients. Our findings reinforce the notion that ACO is a heterogeneous disorder and, as a consequence, it might be unacceptable to offer the same treatment for two related but different conditions.

## Dear editor

We have read with great interest the letter by Kolsum et al. [1] and we fully agree that eosinophilic COPD (e-COPD) patients have distinct characteristics compared to smoking asthmatics (SA) who develop non-fully reversible airflow obstruction. Both entities are commonly encompassed under an umbrella term [2], the so-called Asthma-COPD overlap (ACO), but, in the age of personalized medicine [3], it might be unacceptable to offer the same treatment for two related but different conditions. Studies that focus on identifying ACO’s phenotypes are scarce, but Lange et al. found that individuals with ACO and asthma onset before the age of 40 years have better prognosis than those whose asthma starts after this age [4]. On the other hand, given that asthma and COPD are themselves heterogeneous diseases, one could argue whether it is necessary to define their overlap as a new entity. All these problems could be sorted out by identifying endotypes of obstructive lung disease (OLD) that would allow a personalized approach to therapy. In this regard, we have recently published a study that postulated the extinction of ACO and the use of a Th2 inflammation biomarker to differentiate a pooled population of patients with OLD [5]. With this letter, we would like to provide additional information to support the differentiation between e-COPD and SA.

We have performed a cross-sectional, observational, multicenter study carried out in 23 out-patient clinics from tertiary hospitals in Spain. The details of the design are described elsewhere [5]. Two hundred and ninety-two patients with OLD were included in the study: 94 non-smoking asthmatics, 89 non-eosinophilic COPD, 44 SA and 65 e-COPD. All investigators were asked to prospectively recruit 12 consecutive eligible patients with OLD from their clinics.

Patients were labelled as SA if they had been previously diagnosed with asthma according to GINA guidelines [6] and, after having smoked >20 pack-years, they subsequently developed non-fully reversible airflow obstruction (FEV1/FVC <70% post-bronchodilator). The diagnosis of e-COPD was made in patients who were previously diagnosed with COPD according to GOLD recommendations [7], in the absence of a clinical suspicion for asthma and in the presence of a blood eosinophil count >200 eosinophils/μl.

There were important differences between patients with SA and e-COPD (Table 1). Patients classified as e-COPD were older and significantly more often male. They showed significantly lower post-bronchodilator FEV1 than SA patients (54.49 ± 15.2 vs 65.57 ± 17.5%; *p* = 0.005) and lower DLCO values (64.0 ± 19.9 vs 70.0 ± 17.6%; *p* < 0.001), likely due to a heavier smoking habit (51.8 ± 28 vs 35,1 ± 13.2 pack-year; p < 0.001). On the contrary, SA had more atopic features, more reversibility of airflow obstruction and higher IgE levels. There were no significant differences between the 2 groups with respect to symptoms and exacerbations. Detailed demographic, clinical and functional information is displayed in Table 1. Part of these data have been already published elsewhere [5].

**Table 1:** Demographics, clinical and functional characteristics of patients

Variables^a^	Smoking asthmatics	e-COPD	*P* Value^l^
Number of subjects	44	65	
Age, yrs	59.8 (10.5)	65.6 (10.1)	0.007
Gender (% female)	59.1	18.5	<0.001
BMI (Kg/m^2^)	28.2 (5.2)	28.8 (6.5)	0.91
Pack/yrs^b^	35.1 (13.2)	51.8 (28)	<0.001
Age of onset (yrs)	48.5 (19.1)	53.6 (12.3)	<0.001
SPT^c^ (%)	45.5	23.1	0.003
Rhinitis (%)	51.2	17.5	0.001
Patients with nocturnal symptoms (%)	39.5	9.5	0.001
Comorbidities:	38.6	47.7	0.36
-Arterial hypertension (%) -Diabetes (%)	9.1	23.1	0.26
-Ischemic heart disease (%)	4.5	4.6	0.11
-Heart failure (%)	1.0	7.7	0.26
-Anemia (%)	0	1.5	0.58
-Osteoporosis (%)	18.6	3.2	0.002
-Psychiatric disorders (8%)	18.2	4.6	0.15
-Gastro-esophageal Reflux (%)	35.7	12.7	<0.001
Prebd FEV1^d^ (%)	59.0 (18.3)	50.3 (14.3)	<0.001
Posbd FEV1^e^(%)	65.5 (17.5)	54.5 (15.2)	0.001
PBT^f^ (%)	48.8	30.2	0.001
DLCO^g^ (%)	70.0 (17.6)	64.0 (19.9)	<0.001
Exacerbations^h^	1.00 (1.3)	0.95 (1.3)	0.62
IgE (IU/ml)	112 (4, 1340)	76 (5,2500)	0.005
CAT^i^	13.2 (8.0)	13.4 (7.7)	0.83
ACT^j^	19.5 (4.9)	19.2 (4.8)	0.54

^a^numerical data are expressed as mean (SD) except for IgE and eosinophils which are expressed as median (range); ^b^the pack/years index was calculated in smokers and non-smokers; ^c^prick skin test; ^d^prebronchodilator FEV1; ^e^postbronchodilator test; ^f^rate of patients with positive bronchodilator test; ^g^carbon monoxide diffusing capacity; ^h^number of severe exacerbations during the past 12 months; ^i^COPD Assessment Test; ^j^Asthma Control Test; ^l^statistical significance. *P* values from Student’s t test (continuous normally distributed variables), Mann-Whitney U test (non-normally distributed variables) or Chi-square tests (proportions)

These results are well in accordance with those found by Kolsum et al., and the most remarkable difference is that we were unable to observe any difference between groups in the exacerbation rate in the 12 months prior to study entry, while they found AS to be more prone to suffer exacerbations. To explain this discordance, we must take into account that our patients were fairly well-controlled as assessed by the CAT and ACT questionnaires and the low exacerbation rate.

In order to obtain more detailed information about the underlying inflammatory pattern of OLD patients, we have measured Th-2 characteristic biomarkers such as fractional exhaled nitric oxide (FeNO), blood eosinophils, periostin (DuoSet® ELISA, R&D Systems, Minneapolis, MN, USA) and the concentrations of IL-5 and IL-13 in serum. Additionally, serum levels of cytokines representative of systemic inflammation (TNFα and IL-6) and those associated with a neutrophilic inflammatory response (IL-17 and IL-8) were determined (Merck Millipore®). The levels of these cytokines were similar between SA and e-COPD groups. However, Th2-related biomarkers (periostin, FeNO and blood eosinophils) showed higher median values in e-COPD patients (Table 2). Approximately 49% of e-COPD patients and 30% of SA showed a “Th-2 high” inflammatory pattern (defined as eosinophil count >300 eosinophils/μL in blood or ≥3% in sputum) (*p* = 0.02). Again, these results are consistent with the ones found by Kolsum et al. Interestingly, they observed that only 35.7% of SA patients had blood eosinophil counts ≥300 cells/μl (a similar percentage to our 30%) and only 46% of them showed sputum eosinophil counts ≥3%. What seems clear is that SA forms a more heterogeneous group from the inflammatory point of view- than e-COPD, probably encompassing Th-2 high, neutrophilic and mixed endotypes. This could be explained by the fact that SA definition relies on clinical characteristics (asthma diagnosis and smoking history) whereas e-COPD definition includes a biomarker of Th-2 inflammation. In fact, periostin and FENO were higher in the e-COPD group compared to SA and it would also have been expected to find higher values of Th2-related cytokines (IL-5, IL-13), but we did not perceive statistically significant differences, although a tendency could be observed (Table 2). These discordant results could be justified by an insufficient sample size or by the lack of association between lung and serum biomarkers reported by several authors, both in COPD [8, 9] and asthma patients [10]. It has been shown that the correlation between blood and sputum eosinophilia becomes weaker in those patients with severe asthma and it has been hypothesized that type 2 innate lymphocytes, a steroid-resistant cell, able to produce eosinophilopoietic factors, could explain the persistence of sputum eosinophils in patients with normal blood counts [10]. If such a discrepancy exists, it would not be surprising to find it in other inflammatory mediators such as Th2-related cytokines. A different approach would be to classify ACO patients according to the inflammatory pattern (specific endotypes) in order to evaluate, in a second step, whether it might result in clinical differences. However, we have found poor correlation between the inflammatory markers, which hinders the possibility of defining distinct inflammatory clusters. For example, correlations between IL-5 and IL-13, between IL-5 and blood Eos and between IL-5 and FENO were 0.4, 0.06 and 0.1 respectively in SA patients (0.3, 0.03 and 0–1 in e-COPD patients).

**Table 2:** Differences in the inflammatory profile between the 2 groups

	Smoking asthmatics *n* = 44	e-COPD *n* = 65	*p*-value
IL-13	1.98 (0.23–3.92)	2.18 (0.23–4.20)	0.352
IL-17	7.26 (2.37–16.35)	6.67 (3.12–10.81)	0.142
IL-5	1.63 (0.40–2.77)	1.75 (0.42–2.85)	0.371
IL-6	1.37 (0.42–3.28)	1.43 (0.67–4.12)	0.426
IL-8	9.54 (6.40–14.20)	9.68 (6.25–12.98)	0.652
TNF	3.22 (2.19–4.32)	4.00 (3.19–5.07)	0.740
Periostin	30.8 (23.3–38)	39.7 (30–49.2)	0.005
FENO	19.6 (10–19.9)	24.5 (10–24.6)	<0.001
Blood Eos (cels/μl)	200 (0,1000)	300 (210,940)	<0.001

Numerical data are expressed as median and (interquartile range) except for eosinophils which are expressed as median (range). *P* values from Mann-Whitney U test

*p*-values

It has been largely debated whether asthma and COPD are distinct entities generated by different mechanisms [11] or, on the contrary, they are in fact expressions of one basic disease in which the combined endogenous (host) and exogenous (environmental) factors shape the patient’s clinical profile [12]. Irrespective of that, a current perspective of classification and therapeutic management of OLD patients should take into account the growing knowledge on molecular pathways that has allowed the development of novel therapeutic strategies that target specific components of the underlying inflammatory process. Previous attempts to define ACO [2, 6, 7, 13] do not consider the biological heterogeneity that we, and Kolsum et al., have found, which might potentially lead to inadequate therapeutic approaches. Any useful definition of ACO should offer guidance to make therapeutic decisions, particularly to effectively select who of them can benefit from inhaled corticosteroids treatment (or even anti-Th2 biological drugs in the near future).

In conclusion, our findings reinforce the notion that ACO is a heterogeneous disorder. In fact, OLD is heterogeneous and classical diagnostic categories are unable to fully explain the great complexity of the underlying inflammatory process that ultimately determines the response to treatment. Therefore, we must advance step by step towards a more personalized medicine.
